# Supplementary material for: A fault-intrusion-tolerant system and deadline-aware algorithm for scheduling scientific workflow in the cloud
Source: PeerJ Comput Sci. 2021 Nov 2;7:e747. doi: 10.7717/peerj-cs.747 (PMC8576554; doi:10.7717/peerj-cs.747)
Supplement: Supplemental Information 6 [file peerj-cs-07-747-s006.zip › Myproject1.1/docs/org/cloudbus/cloudsim/lists/ResCloudletList.html]

ResCloudletList


JavaScript is disabled on your browser.


- Overview
- Package
- Class
- Use
- Tree
- Deprecated
- Index
- Help

- Prev Class
- Next Class

- Frames
- No Frames

- All Classes

- Summary:
- Nested |
- Field |
- Constr |
- Method

- Detail:
- Field |
- Constr |
- Method


org.cloudbus.cloudsim.lists

## Class ResCloudletList

- java.lang.Object
- - org.cloudbus.cloudsim.lists.ResCloudletList

- ---

    

  ```
  public class ResCloudletList
  extends java.lang.Object
  ```

  ResCloudletList is a collection of operations on lists of ResCloudlets.

  Since:
  :   CloudSim Toolkit 2.0

- - ### Constructor Summary

    Constructors

    Constructor and Description

    `ResCloudletList()`
  - ### Method Summary

    Methods

    | Modifier and Type | Method and Description |
    | --- | --- |
    | `static <T extends ResCloudlet>  ResCloudlet` | `getByIdAndUserId(java.util.List<T> list, int cloudletId, int userId)` Returns a given Cloudlet. |
    | `static <T extends ResCloudlet>  int` | `getPositionById(java.util.List<T> cloudletList, int id)` Returns the position of the cloudlet with that id, if it exists. |
    | `static <T extends ResCloudlet>  int` | `indexOf(java.util.List<T> list, int cloudletId, int userId)` Finds the index of a Cloudlet inside the list. |
    | `static <T extends ResCloudlet>  boolean` | `move(java.util.List<T> listFrom, java.util.List<T> listTo, T cloudlet)` Move a ResCloudlet object from this linked-list into a specified one. |

    - ### Methods inherited from class java.lang.Object

      `clone, equals, finalize, getClass, hashCode, notify, notifyAll, toString, wait, wait, wait`

- - ### Constructor Detail


    - #### ResCloudletList

      ```
      public ResCloudletList()
      ```
  - ### Method Detail


    - #### getByIdAndUserId

      ```
      public static <T extends ResCloudlet> ResCloudlet getByIdAndUserId(java.util.List<T> list,
                                                         int cloudletId,
                                                         int userId)
      ```

      Returns a given Cloudlet. This method needs a combination of Cloudlet Id and User Id because
      each Cloud Users might have exactly same Cloudlet Ids.

      Parameters:
      :   `cloudletId` - a Cloudlet Id
      :   `userId` - an User Id
      :   `list` - the list

      Returns:
      :   the Cloudlet.

      Throws:
      :   `java.lang.IndexOutOfBoundsException` - - if a Cloudlet with specified Id and user id is not in the
          list.


    - #### indexOf

      ```
      public static <T extends ResCloudlet> int indexOf(java.util.List<T> list,
                                        int cloudletId,
                                        int userId)
      ```

      Finds the index of a Cloudlet inside the list. This method needs a combination of Cloudlet Id
      and User Id because each Cloud User might have exactly the same Cloudlet Id.

      Parameters:
      :   `cloudletId` - a Cloudlet Id
      :   `userId` - an User Id
      :   `list` - the list

      Returns:
      :   the index in this list of the first occurrence of the specified Cloudlet, or
          `-1` if the list does not contain this Cloudlet.


    - #### move

      ```
      public static <T extends ResCloudlet> boolean move(java.util.List<T> listFrom,
                                         java.util.List<T> listTo,
                                         T cloudlet)
      ```

      Move a ResCloudlet object from this linked-list into a specified one.

      Parameters:
      :   `listFrom` - the list from
      :   `listTo` - the list to
      :   `cloudlet` - the cloudlet

      Returns:
      :   **true** if the moving operation successful, otherwise return **false**


    - #### getPositionById

      ```
      public static <T extends ResCloudlet> int getPositionById(java.util.List<T> cloudletList,
                                                int id)
      ```

      Returns the position of the cloudlet with that id, if it exists. Otherwise -1.

      Parameters:
      :   `cloudletList` - - the list of cloudlets.
      :   `id` - - the id we search for.

      Returns:
      :   - the position of the cloudlet with that id, or -1 otherwise.


- Overview
- Package
- Class
- Use
- Tree
- Deprecated
- Index
- Help

- Prev Class
- Next Class

- Frames
- No Frames

- All Classes

- Summary:
- Nested |
- Field |
- Constr |
- Method

- Detail:
- Field |
- Constr |
- Method
